# Are Meat Quality Traits and Sensory Attributes in Favor of Slow-Growing Chickens?

**DOI:** 10.3390/ani10060960

**Published:** 2020-05-31

**Authors:** Erika Pellattiero, Giulia Tasoniero, Marco Cullere, Elizabeth Gleeson, Gabriele Baldan, Barbara Contiero, Antonella Dalle Zotte

**Affiliations:** 1 Department of Animal Medicine, Production and Health, University of Padova, Agripolis, viale dell’ Università, 16, 35020 Legnaro (P.D.), Italy; erika.pellattiero@unipd.it (E.P.); giulia.tasaniera@usda.gov (G.T.); elizabethyvonne.gleeson@studenti.unipd.it (E.G.); barbara.contiero@unipd.it (B.C.); antonella.dallezotte@unipd.it (A.D.Z.); 2 Agricultural High School “San Benedetto da Norcia”, Via delle Cave 172, 35136 Padova, Italy; sbalengo10@gmail.com

**Keywords:** chicken genotype, Padovana chicken, Polverara chicken, haem iron, sensory analysis, consumer preference

## Abstract

**Simple Summary:**

Considering the food product meat, consumers are increasingly interested in nutritional quality, and also in extrinsic attributes such as animal welfare and environmental sustainability. In this regard, local chicken breeds can represent an opportunity to exploit alternative markets and to satisfy the above-mentioned consumer expectations. With this in mind, the present study compared quality and sensory characteristics of meat obtained from three different chicken genotypes: a conventional broiler Hybrid, and two endangered indigenous Italian breeds, i.e., Padovana and Polverara. Results indicated that an unknown meat product with peculiar quality and sensory characteristics might not be appreciated by inexperienced consumers. This is because quality perception is influenced by many factors such as specific cultural background and familiarity with a product’s sensory attributes. Therefore, the development of appropriate marketing strategies is a crucial step to inform consumers about product characteristics, thus allowing to exploit a promising market potential which, in turn, would also allow chicken breed conservation.

**Abstract:**

The present study compared certain quality features and sensory attributes of the meat obtained from three different chicken genotypes: two endangered slow-growing indigenous breeds (Padovana: PAD, Polverara: POL) and a commercial broiler (Hybrid). Chickens (*n* = 42/genotype) were slaughtered at their commercial slaughter age: 183 days for PAD and POL chickens, and 35 days for the Hybrid. Breast and leg meat were dissected and used for qualitative evaluations. Results highlighted that genotype significantly affected final breast weight, with Hybrid showing higher values than both indigenous chickens. Conversely, PAD and POL meat was instrumentally tougher and richer in haem iron compared to the Hybrid. The sensory analysis conducted by a trained panel showed that the textural aspects and metallic flavor perception of PAD and POL meat differed from that of the Hybrid. These aspects were probably responsible for the lower overall acceptability of the PAD and POL meat compared to the Hybrid, as expressed by the consumer panel. A key step in making PAD and POL meat acceptable to consumers and stimulating its market seems thus the development of appropriate marketing strategies to transform product peculiarities in strength points.

## 1. Introduction

Meat and meat products have historically played an important role in the human diet. Among meat species, chicken represents the most consumed meat type worldwide. This is possibly thanks to the lack of cultural prejudices related to its consumption, its mild taste, favourable textural attributes, and overall nutritional profile including a low-fat content [1,2]. Consequently, the broiler chicken sector has grown to the point where it has become one of the most intensive farming systems in the world, characterized by high stocking densities, indoor rearing, and fast-growing hybrids obtained through genetic selection which has made it possible to slaughter birds within 40 days of age [3,4]. Fast-growing commercial hybrids are characterized by improved feed efficiency and increased carcass and breast yields [5]. Moreover, the size of meat cuts makes it possible to exploit them in the creation of several processed products which meet the target “cooking easiness” of modern consumers [6]. These developments in the poultry meat sector have contributed to a loss of genetic material and, consequently, to a loss of biodiversity. This could potentially lead to the extinction of numerous indigenous chicken breeds [7].

Nowadays, a part of consumers has exhibited an increasing interest in meat products, including fresh chicken meat, characterized by different intrinsic and extrinsic quality attributes in respect to those obtained from conventional production [8]. Such attributes include nutritional characteristics, product safety and the farming system, which should respond to the criteria of environmental sustainability and high animal welfare standards, including the possibility for the intended animal species to satisfy its behavioral repertoire [9]. Consequently, the increasing awareness of consumers towards the above-mentioned topics is strongly pushing the development of new markets which offer unconventional meat and meat products [8]. In this sense, fast-growing chicken hybrids cannot be utilized in alternative farming systems, whereas slow-growing chicken breeds are perfectly suitable. This is because they are rustic and have a slower and alternatively proportioned muscle and organ growth. This makes them capable of adapting to environmental changes and to be not dependent on strict nutritional requirements, thus easily exploiting diverse diets [10]. Successful commercial examples of chicken meat obtained from slow-growing chicken breeds can be observed in different countries, including France, the UK, the Netherlands, and Germany [11].

In the Veneto region of Italy there are different local chicken breeds that have distinct meat quality characteristics, most of which are at risk of extinction and many are in specific conservation programs. Among them, the Padovana and Polverara chickens are dual purpose breeds characterized by a slow growth rate, a high resistance to diseases, and good environmental adaptability, therefore having the potential to be used in extensive production systems [12,13,14,15,16,17]. 

Recent studies by Tasoniero et al. [18] and Dalle Zotte et al. [19,20,21] investigated the productive performance, slaughter yield, and meat quality of Padovana and Polverara breeds, with the objective to characterize their unique meat quality properties and potential in the poultry industry, and showed that these slow-growing chickens can effectively be utilized in alternative production systems. From a nutritional point of view, the meat obtained from these two breeds has a high protein and low lipid contents with a favorable fatty acid profile [14,19]. Furthermore, a study considering the proximate composition and amino acid profile of Polverara breast meat highlighted the superior quality traits compared to conventional chicken meat [21].

The last step to characterize the meat traits of Padovana and Polverara breeds relates to the evaluation of specific meat quality features and sensory profile. Previous research showed that the meat is uniquely redder than conventional chicken meat [18], thus suggesting a possible significant content of haem iron. Furthermore, sensory quality is of fundamental importance for future marketing purposes as it drives consumers’ product choice at purchase [22]. For this reason, a panel as well as a consumer evaluation is necessary. 

Based on the above-mentioned premises, the present research study compared specific meat quality traits and the sensory profile of the meat of the Padovana and Polverara slow-growing chicken breeds to those of a conventional broiler hybrid. 

## 2. Materials and Methods

### 2.1. Experimental Groups

This research study was conducted *post mortem* and no ethical approval by the Ethical Committee was therefore requested. At 183 days of age, 42 Padovana and 42 Polverara chickens of both sexes were randomly selected from the broiler unit at the Agricultural Professional High School ‘Duca degli Abruzzi’ (Padova, Italy) and slaughtered at a commercial abattoir [18]. On the same day and in the same abattoir, the carcasses of 42, 35-day old, fast-growing commercial hybrid chickens (Hybrid) were randomly selected. After chilling (precooling at 5 °C for 60 min, followed by chilling at 0 °C for 90 min), carcasses were transported to the Department of Animal Medicine, Production and Health (MAPS) at the Padova University (Italy) and dissected. Breasts (*Pectoralis major*) and legs (thigh and drumstick) were excised, individually tagged, vacuum packed, and frozen at −40 °C. As a result, three experimental groups were obtained: Padovana (PAD), Polverara (POL), and Hybrid. Slow-growing chicken management, slaughter procedure, and processing were previously described in the paper by Tasoniero et al. [18]. 

### 2.2. Physical and Chemical Analyses

Water holding capacity (thawing, cooking, and total losses) and Warner–Bratzler shear force (WBSF) were assessed on 24 left breast fillets per group (*n* = 72). Breast cuts were thawed overnight at 4 °C, then weighed, and thawing loss was calculated as percentage of the frozen weight. Samples were then individually vacuum-sealed in polypropylene bags and cooked in a water bath set at 80 °C, until a core temperature of 78 °C was reached. Once cooked, samples were cooled by immersing bags in cold water, then weighed again to compute cooking loss, whereas total loss was calculated as percentage of the initial weight. Subsequently, WBSF data were collected from each breast: 5 cylindrical cooked meat pieces (1.25-cm diameter × 2-cm length) were obtained by coring the meat parallel to the orientation of the muscle fibers, by using a mechanical coring device [23]. Cylinders of cooked meat were cut, as described by Sams et al. [24] with a Warner–Bratzler cell (100 kg load cell, 2-mm/s crosshead speed) fitted on a TA-HDi Texture Analyzer (Stable Macro System, London, UK). Haem iron content was determined on 12 left breast fillets per group (*n* = 36) and on 12 left legs per group (*n* = 36), chopped, and ground using a Retsch Grindomix GM 200 (10 s at 7000 g). The remaining legs were used for different analytical purposes [21]. Samples were analyzed following the method by Hornsey [25] and read with the absorbance at 640 nm; results were expressed as mg/kg of meat.

### 2.3. Descriptive Sensory Analysis

A descriptive sensory analysis was performed as defined by the International Organization for Standardization (ISO) 13299 (2003) [26] to identify the possible differences among the three genotypes. In total, 102 right breasts (34 per group) were offered to a panel comprised of 17 trained assessors and took place at the “Istituto per la qualità e le Tecnologie Agroalimentari”, Veneto Agricoltura (Thiene, Vicenza, Italy). With the objective of familiarizing them with the sensory attributes, the panellists took part in a three-week orientation training session. During the training session, purchased and frozen chicken breasts were served and evaluated as reference, allowing panelists to identify descriptors and assimilate intensity scores [26]. For the analysis, intact frozen breasts were thawed, given a random three-digit code, and grilled on a cooking plate (model GR6010 XL Health Comfort, 2400 Watt; Rowenta, Erbach, Germany) to an internal temperature of 78 °C. From each breast, two 1.9 × 1.9 cm cubes were obtained following the diagram by Lyon and Lyon [27] and served while still warm. Each assessor evaluated two breasts per group and fillets were served in a randomized order with a 15-min recess between each evaluation. Evaluations took place in a testing room with temperature of 21 °C, neutral-colored walls and furniture, and standard lighting conditions. Permanent individual testing booths were equipped with plastic cutlery, a plastic dish and glass, expectorant cup, water, unsalted crackers, and six paper ballots (one per sample). The list of sensory descriptors is presented in Table 1. The descriptors were scored from 0 (the lowest value) to 10 (the highest value) on a continuous scale. In addition, panelists were asked to indicate the presence of off-odors and off-flavors perceived. 

### 2.4. Consumer Sensory Analysis

A consumer sensory analysis was performed on 42 right legs per group (*n* = 126 in total). Chicken legs were thawed overnight at +4 °C, deboned, chopped, and ground using a Retsch Grindomix GM 200 (10 s at 7000 g). The ground meat was then used to form 162 meatballs per group, weighing 8–10 g each. They were then vacuum-packed and cooked as indicated below. Consumers were recruited among students (14–20 years old, 70% of the panelists) and personnel (35–61 years old, 30% of the panelists) from the Agricultural Professional High School “Duca degli Abruzzi” (Padova). A total of 150 people were involved in the analysis. Screening consisted of a questionnaire aimed at the evaluation of age, health condition, and level of acceptance and consumption of chicken meat. As a preference test was administered, no experience in sensory analysis or training was required. The test was carried out in a single morning and consisted of five tasting sessions of 30 people each. Each session, assessors received instructions on how to fill the paper ballot and were equipped with plastic cutlery and a glass, water, unsalted crackers, a pencil, and paper ballot. Meatballs were cooked in-bag in a water bath set at 80 °C for 10 min and served still warm to the consumers, without added salt or seasoning. The samples from the three experimental groups were served at the same time. For each sample, consumers were asked to express their judgment on visual appearance, chicken odor, chicken flavor, juiciness, greasiness, and overall product acceptability, indicating for each attribute whether the sample was either extremely unacceptable, unacceptable, moderately unacceptable, moderately acceptable, acceptable, or extremely acceptable. A system of evaluation with scores from 1 (extremely unacceptable) to 6 (extremely acceptable) was considered for each attribute.

### 2.5. Statistical Analysis

Experimental data were analyzed by SAS (2004 version 9.4) [28] statistical software package for Windows. Breast weight and physical and chemical traits were evaluated using one-way ANOVA and processed by choosing a general linear model that considered genotype as fixed effect (General Linear Models Procedure—PROC GLM). Using the same fixed effect, a mixed model (PROC MIXED) was performed to evaluate descriptive sensory data with panelists as random effect; a single tasting was considered as experimental unit.

Least square means were calculated and post hoc pairwise comparisons among levels were applied using Bonferroni adjustments. Where data were expressed as counts (number of samples with rancid odor, rancid and metallic flavor) a chi-square approach was applied to test the genotype effect. The *p* < 0.05 was considered as significance level.

Consumer’s preference data were analyzed by different general linear models (PROC MIXED) considering genotype, consumer’s age, consumer’s gender, and consumer’s frequency of chicken meat consumption as fixed effects. Panelist was considered as random effect and a single repeated tasting was considered as experimental unit. Least square means were calculated, post hoc pairwise comparisons among levels were applied using Bonferroni adjustments, and *p* < 0.05 was considered as significance level. As consumer’s gender and frequency of chicken meat consumption were not significant, the specific tables were not included in the manuscript, but they are available as Supplementary Material (Tables S1 and S2). 

A discriminant analysis was also performed on descriptive sensory and consumer’s sensory data. The analysis extracted the first two principal components and gave the preference scores most correlated with them. The analysis also highlighted the variables that were more discriminant among genotypes.

## 3. Results

### 3.1. Physical Parameters and Haem Iron Content

Results depicted in Table 2 show that the genotype (Hybrid, PAD, and POL) significantly affected the physical meat quality traits of all the considered parameters, except total loss. Chicken breast was significantly heavier for Hybrid compared to PAD and POL groups (*p* < 0.0001). Thawing loss showed higher values for PAD and POL breasts compared to Hybrid (*p* < 0.0001). Conversely, cooking loss was the highest for the Hybrid group (*p* = 0.0019). Despite the latter results, total loss was similar for all treatments. Meat toughness, expressed as Warner–Bratzler shear force (WBSF), was the highest for PAD and POL meat (*p* = 0.0004). 

The genotype had a significant effect also on the haem iron content of breast and leg meat cuts (Table 3): Slow-growing chickens (PAD and POL) exhibited a > 3 times higher content compared to Hybrid chickens (*p* < 0.0001). The significance and the magnitude of the difference found for haem iron content was similar for the two different meat cuts when comparing the two slow-growing chicken groups to the Hybrid group. Furthermore, leg meat of POL chickens was, in absolute terms, the richest in haem iron as it displayed a significantly higher value also compared to the PAD group.

### 3.2. Descriptive Sensory Analysis and Off-Attribute Prevalence

Results concerning breast meat sensory attributes as a function of the different genotypes are presented in Table 4 and Table 5. In general, genotype exhibited a mild effect on the considered descriptors as most of them remained unaffected. The two exceptions were toughness (*p* = 0.0236) and fibrousness (*p* = 0.0275): these two sensory attributes were higher in the breast meat of PAD group compared to Hybrid, with POL showing intermediate values.

Data regarding breast meat off-attributes (rancid odor, rancid flavor, and metallic flavor) prevalence as a function of the genotype are shown in Table 5. Genotype influenced metallic flavor perception (*p* = 0.002), whose values were higher for PAD and POL groups compared to the Hybrid group. Conversely, rancid odor and flavor did not show statistically significant differences, even though the prevalence of these two off-attributes was observed numerically higher in the Hybrid group compared to the others. 

### 3.3. Consumer Preference Analysis

Table 6, Table 7 and Table 8 show the results of the consumer preference analysis test. The genotype (Hybrid, PAD, and POL) affected all the considered sensory attributes evaluated by the consumer panel (Table 6). In most cases (visual appearance, juiciness, greasiness, overall acceptability), the best scores were assigned to the leg meatballs of the Hybrid chicken. Regarding chicken odor, Hybrid and PAD meat showed similar values, which were higher than POL group (*p* = 0.0251). For the variables’ chicken flavor (*p* < 0.0001) and overall acceptability (*p* < 0.0001), the groups were scored as follows: Hybrid > PAD > POL.

Results presented in Table 7 highlight that consumer age was a key factor in determining the scores assigned to the sensory attributes of chicken meat. In fact, with the exception of greasiness (*p* = 0.0532), all other attributes evidenced that mature panellists tended to assign higher scores compared to younger panelists; this was observed for visual appearance (*p* = 0.0245), chicken odor (*p* = 0.0073), chicken flavor (*p* = 0.0325), juiciness (*p* = 0.0113), and overall acceptability (*p* = 0.0022). 

When looking at the results of the same analysis presented in Table 6, but considering each chicken genotype separately (Table 8), a similar trend emerged. In this case as well, the consumer’s age was a key factor for the assignment of the sensory scores and most traits were significantly affected by the considered effect, with personnel always assigning higher scores than students. For the Hybrid group, the traits affected by consumer age were visual appearance (*p* = 0.004), chicken odor (*p* = 0.026), chicken flavor (*p* = 0.015), juiciness (*p* = 0.004), and overall acceptability (*p* = 0.006). For meat of the PAD group, the variables which were affected by the tested effect were visual appearance (*p* = 0.003), chicken odor (*p* = 0.025), juiciness (*p* = 0.016), greasiness (*p* = 0.012), and overall acceptability (*p* = 0.023). In the case of the POL meat, three out of six sensory attributes were affected by consumer age: chicken odor (*p* = 0.002), juiciness (*p* = 0.018), and greasiness (*p* = 0.043). 

**Figure 1:**
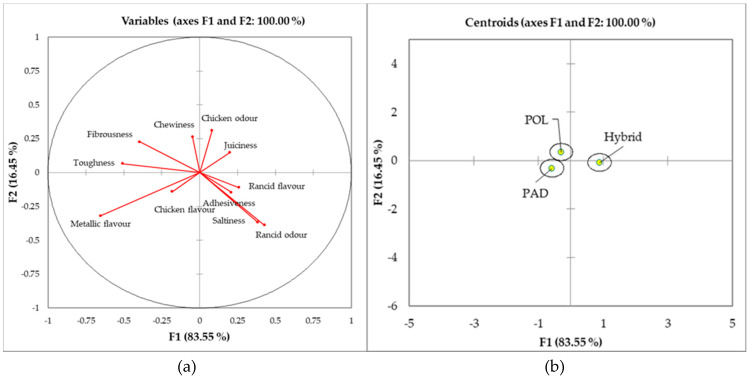
(**a**,**b**) Discriminant analysis performed on the sensory data of the trained panel. Hybrid: Hybrid chicken. PAD: Padovana slow-growing breed. POL: Polverara slow-growing breed.

**Figure 2:**
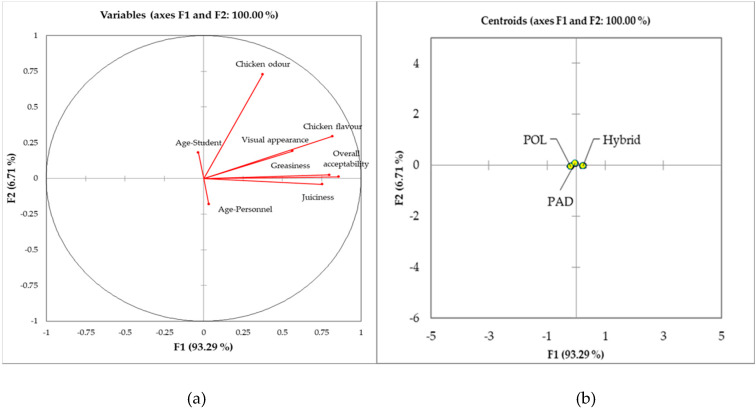
(**a**,**b**) Discriminant analysis performed on the sensory data of the consumers’ panel. Hybrid: Hybrid chicken. PAD: Padovana slow-growing breed. POL: Polverara slow-growing breed.

Figure 1a,b and Figure 2a,b show the results of the discriminant analysis performed on the sensory panel data for panelists and consumers, respectively. The analysis highlighted the preference scores most correlated with the first two principal components and discrimination among genotypes (PAD vs. POL vs. Hybrid). For the sensory analysis performed by trained panellists (Figure 1a), the first component, which accounted for the 84% of the total variance, highlighted positive correlations mainly with rancid odor (0.43) and saltiness (0.38), but also with rancid flavor (0.26), adhesiveness (0.21), and juiciness (0.20). The main negative correlations, instead, were observed for metallic flavor (−0.65), toughness (−0.51), and fibrousness (−0.40). The second principal component, which accounted for the 16% of the total variance, was mainly positively correlated with chicken odor (0.31), chewiness (0.27), and fibrousness (0.23). With regards to the negative correlations, rancid odor (−0.39), saltiness (−0.37), and metallic flavor (−0.32) were the main variables. In general, however, centroids’ position indicated that panelists had a limited, nonsignificant ability to discriminate among the considered genotypes (Figure 1b); in fact, only a small distance of the Hybrid group compared to PAD and POL ones could be noticed.

Figure 2a,b reports the results of the discriminant analysis performed on the sensory consumer preference data. Students and personnel were considered as dichotomous variables. Therefore, each of them was placed in opposite positions inside the quadrants. In particular, the graph aimed at assessing if the considered sensory variables were perceived differently by the two groups of consumers, which was expressed by the relative distance of each sensory descriptor from the Age-Student/Age-Personnel points. Results showed that personnel seemed overall less sensitive compared to students for most traits; the only exception was juiciness, which was better perceived by personnel. The fact that almost all sensory variables were placed in the same quadrant showed that descriptors were all linked together, but also that a distinction among the three genotypes was not possible. This is particularly evident in the Figure 2b, where the centroids of the three experimental groups are depicted.

## 4. Discussion

The Padovana (PAD) and Polverara (POL) are local Italian chicken breeds, which have scarcely been selected for growth traits, thus showing moderate breast weights. Selective efforts instead have been mainly directed towards fixing certain morphological traits, in accordance with the “Registry of Italian Indigenous Chicken Breeds” (Ministerial Decree No. 19536 of 01.10.2014) [29]. Results of the present study showed that the average breast weight for both local breeds slaughtered at six months of age (i.e., their commercial slaughter age) was 117 g. This value is extremely low compared to conventional broiler hybrids, but also inferior compared to a previous research [18], which considered both male and female Padovana and Polverara chickens (average breast weight = 193 g). This finding indicates that the poor genetic selection for growth parameters in these local breeds can result in inhomogeneous productive outcomes, possibly being dependent on the considered chicken stock. This is exacerbated by the farming system, which exposes chickens much more to environmental changes compared to conventional farming systems, where environmental parameters are strictly controlled. 

With the exception of bound water, which makes up for a minor proportion of total water in the muscle, immobilized and free water are influenced by *post mortem* factors. To this regard, stress is known to play a key role: short-term stress before slaughter increases in vivo metabolic rate, but it can also influence the *peri mortem* metabolism of the animal. As a result, the rate in pH decline and ultimately the water holding capacity of meat can be affected. The susceptibility of farmed animals to stress factors is known to vary also among genotypes [30]. In the present experiment the genetic source could partly explain why meat of PAD and POL chickens showed a different pattern for water loss compared to the meat of the conventional Hybrid: for the first two breeds, the loss of water was more prevalent during thawing, whereas the other genotype lost more water during cooking. However, ultimately this was not a relevant factor for the total water loss, which was comparable among treatments. Together with moisture loss, the WBSF of meat is known to be affected by several intrinsic factors related to the animal itself i.e., age, muscle energy metabolism, collagen content, maturity of the connective tissue [31,32]. Therefore, it was not surprising that breast meat of PAD and POL chickens was instrumentally tougher than that of the conventional Hybrid chicken: the two slow-growing breeds were slaughtered at 6 months of age, which roughly corresponds to at least 3 times the slaughter age of conventional broiler hybrids. In the case of the present experiment, Hybrid chickens were slaughtered at 35 days of age. 

The older age of PAD and POL chickens compared to the Hybrid, together with the diverse locomotor pattern, is probably the reason behind the >3 times higher haem iron content of PAD and POL meat compared to the Hybrid, independently of the considered meat cut. In absolute value, haem iron content of PAD and POL meat was found to be similar to that of ostrich and horse meat fillet, which are among the best sources of this element [33]. PAD and POL chickens easily exploit the whole surface that is available for them within the rearing paddock: they walk, explore, and scratch for feed. They sometimes perform dust bathing and, if available, they perch on trees during the night. All these behavioral patterns, typical of the repertoire of the ancestor of the domestic chicken, i.e., the red jungle fowl, are also typical of chicken breeds reared under free-range conditions [34,35]. This behavioral pattern reflects on the metabolism of muscle fibers and thus on the proportion among muscle fiber types as a response to muscle’s function and metabolism [36]. Haem iron content is higher in muscles characterized by a prevalent oxidative metabolism, which is the case of PAD and POL breeds, but not that of broiler chickens. In fact, in the last, the genetic selection pushed for volumetrically bigger muscle fibers (hypertrophy) and for a higher density of fast-twitch fibers, which are characterized by a prevalent glycolytic metabolism [4]. Furthermore, it should also be considered that the great muscle growth and thus weight of Hybrid chickens, combined with farming density and the lack of environmental enrichment, strongly limit their locomotor activity compared to slow-growing chickens reared under extensive conditions.

To the authors’ knowledge, no previous studies have been conducted on the descriptive sensory analysis and consumer sensory analysis for the two slow-growing chicken breeds that were considered in the present study. Human sensory evaluation of food is a complex process which is affected by people’s different experience towards it and also depends on their specific cultural background [37]. Trained assessors typically perform objective sensory analysis and, therefore, remain the most appropriate tool to perceive and explain possible differences among treatments [38]. Consumers, however, even if they are not trained to discriminate among different food-related sensory attributes, represent the final market goal, i.e., every food product is produced to meet consumers’ demands and acceptance [39]. 

Results of the present experiment showed that trained assessors were able to detect only a few sensory differences among tested treatments, i.e., toughness and fibrousness attributes, which did not allow discriminating well among the three genotypes. Both descriptors were found to be higher for the PAD and POL breast meat compared to the Hybrid, which could mainly be explained by the higher slaughter age of the two slow-growing chickens. In fact, meat toughness and fibrousness perception is related, together with the thickness of the perimysium, to its total collagen content [32]. Even if collagen content was not considered in the present experiment, it is widely accepted that it is influenced by genotype, rearing system, and age at slaughter [31]. Specifically referring to the rearing technique, outdoor system, which is used for these local breeds, probably contributed to obtaining tougher and firmer meat as a consequence of the increased physical activity and build-up of collagen tissue. In this sense, sensory results supported the observed WBSF instrumental values, which were as expected, as toughness and fibrousness are typically positively correlated to shear force [40]. The fact that most sensory attributes were not influenced by genotype is in accordance with some literature which compared the sensory characteristics of meat derived from slow-growing and fast-growing chickens in regards to odor, taste, juiciness, and overall acceptability [31]. Other research instead showed that descriptive sensory analysis can effectively distinguish meat coming from different chicken genotypes [41,42]. Regarding the off-attributes’ perception, metallic flavor was found to be more intensely perceived in the meat of PAD and POL breeds compared to the Hybrid chicken, which can be directly linked to the observed differences in the haem iron content of meat. 

When buying a food product, i.e., meat, consumers are generally not aware of its real quality attributes. They tend to form expectations based on available cues, which then affect purchase behavior. Among the many dimensions that they use to evaluate a food product, it is undoubtful that sensory experiences play a pivotal role [37,43]. Considering meat sensory traits, the visual aspect exerts the first relevant function in orienting consumers’ choice [44]. It is also well established that when consumers are not used to consume a certain type of food, they generally dislike it [6]. From previous research, it emerged that the meat of PAD and POL chickens is characterized by different physical and chemical features compared to that of conventional broiler chickens [20,21], being particularly dark and with a marked redness, and with a very low fat content. Having all the above-mentioned considerations in mind, it is easy to perceive why the meat of PAD and POL chickens was penalized by consumers in terms of sensory evaluation compared to the Hybrid. 

These findings highlight that it is necessary to develop appropriate marketing strategies to promote the consumption of meat from PAD and POL chickens, as well as for other indigenous breeds. In fact, it was demonstrated that by exploiting the extrinsic attributes of meat, i.e., free-range/welfare-friendly/local/autochthonous (product and process characteristics), it is possible to have a large impact on consumer choice, increasing product quality expectations [45]. A demonstration of the market success of this promotion strategy comes from the French “Label Rouge” (red label) sign of quality, complementary of a commercial label: many regional producer-oriented associations market, under a single label, their own products. Moreover, a third-party certification program guarantees specific quality standards. This French sign of quality has one flagship product, poultry meat, which is obtained from free-range, slow-growing chickens and whose marketing success is specifically based on proven meat sensory attributes and food safety. Economically, poultry meat sold under this label accounted for 30% of the French poultry market in 2011 and the price of Label Rouge poultry meat reached two times that of conventional chicken meat [46].

Another technical consideration that arose from the sensory evaluation of chicken meat conducted by consumers was that the age of panelists was a relevant factor in assigning sensory scores, with older people giving higher scores than younger people for all genotypes. Previous literature showed that older consumers exhibit different texture perception and aromatic thresholds compared to younger consumers as a result of a progressive decline in olfaction and taste sensitivity [38]. This would hypothetically make them less skeptical than young consumers in judging an unknown food product. Other studies, however, demonstrated that age-related decline in senses’ acuity is compensated by a longer exposure to different aromatic compounds in older consumers, which often helps them to differentiate between subtle taste differences better than younger people, and by the fact that they are generally more predisposed to try, learn, and, thus, accept new meat products compared to younger consumers [47,48]. Finally, coherently to what was observed in the present study, also previous research demonstrated that consumers are often unable to show clear discrimination between different products and genotypes [49]. 

## 5. Conclusions

From this research study it emerged that, at present, the peculiar quality attributes of meat obtained from Padovana and Polverara chicken breeds could be an obstacle to make this product acceptable to consumers. This is probably a result of its darker/redder color, metallic flavor, and textural characteristics. However, these elements could also be considered a strength point in developing appropriate strategies for product marketing. Furthermore, the firm texture of the meat and its colorimetric characteristics make it easily distinguishable from that of conventional broiler chickens. Another strength point is the farming system, which is extensive and allows chickens to express their behavioral repertoire. These are all characterizing elements that could be exploited for marketing purposes and to create niche/alternative markets, aimed at reaching consumers who are sensitive to the above-mentioned features. 

## Figures and Tables

**Table 1:** List of the sensory attributes and scores.

Attributes	Scores	
	0	10
Chicken odor	Not perceived	Extremely strong
Chicken flavor	Not perceived	Extremely strong
Saltiness	Not salty	Extremely salty
Toughness	Extremely tender	Extremely hard
Fibrousness	Not fibrous	Extremely fibrous
Chewiness	Not chewy	Extremely chewy
Juiciness	Not juicy	Extremely juicy
Adhesiveness	Not adhesive	Extremely adhesive

**Table 2:** Effect of genotype (Hybrid, PAD, POL) on breast weight, water holding capacity (thawing, cooking, and total loss), and Warner–Bratzler shear force.

Traits	Genotype			RSD ^1^	*p*-Value
	Hybrid	PAD	POL		
No. of samples	24	24	24		
Breast weight, g	410 ^A^	114 ^B^	119 ^B^	51.8	<0.0001
Thawing loss, %	6.52 ^B^	12.5 ^A^	10.3 ^A^	4.23	<0.0001
Cooking loss, %	29.3 ^Aa^	26.2 ^ABb^	25.1 ^B^	3.99	0.0019
Total loss, %	35.8	38.2	35.4	7.13	0.3724
Warner-Bratzler shear force, N	17.0 ^B^	21.1 ^A^	20.6 ^A^	3.62	0.0004

Hybrid: hybrid chicken. PAD: Padovana slow-growing breed. POL: Polverara slow-growing breed. ^A,B^ Means in the same row with different superscript letters differ for *p* < 0.01. ^a,b^ Means in the same row with different superscript letters differ for *p* < 0.05. ^1^ Residual standard deviation.

**Table 3:** Effect of genotype (Hybrid, PAD, POL) on haem iron content of breast and leg meat.

Haem Iron	Genotype			RSD ^1^	*p*-Value
	Hybrid	PAD	POL		
No. of samples	12	12	12		
Breast haem iron (mg/kg)	1.71 ^B^	5.82 ^A^	5.73 ^A^	0.84	<0.0001
Leg haem iron (mg/kg)	4.25 ^B^	11.8 ^Ab^	16.2 ^Aa^	4.19	<0.0001

Hybrid: hybrid chicken. PAD: Padovana slow-growing breed. POL: Polverara slow-growing breed. ^A,B^ Means in the same row with different superscript letters differ for *p* < 0.01. ^a,b^ Means in the same row with different superscript letters differ for *p* < 0.05. ^1^ Residual standard deviation.

**Table 4:** Descriptive sensory analysis scores of breast meat according to genotype (Hybrid, PAD, POL).

Attributes	Genotype			RSD ^1^	*p*-Value
	Hybrid	PAD	POL		
No. of breast samples	34	34	34		
Chicken odour	5.59	5.35	5.60	0.19	0.1926
Chicken flavour	5.32	5.42	5.42	0.17	0.7638
Saltiness	4.63	4.37	4.35	0.34	0.1139
Toughness	5.10 ^b^	5.69 ^a^	5.52 ^ab^	0.18	0.0236
Fibrousness	5.41 ^b^	5.92 ^a^	5.83 ^ab^	0.17	0.0275
Chewiness	5.24	5.52	5.51	0.28	0.4326
Juiciness	4.92	4.63	4.84	0.19	0.3151
Adhesiveness	4.94	4.82	4.75	0.14	0.5752

Hybrid: hybrid chicken. PAD: Padovana slow-growing breed. POL: Polverara slow-growing breed. ^a,b^ Means in the same row with different superscript letters differ for *p* < 0.05. ^1^ Residual standard deviation.

**Table 5:** Breast meat off-attributes’ prevalence according to genotype (Hybrid, PAD, POL).

Off-Attributes	Genotype			χ^2^	*p*-Value
	Hybrid	PAD	POL		
Rancid odor, %	11.8	2.94	0.00	5.47	0.065
Rancid flavor, %	8.82	2.94	2.94	1.68	0.431
Metallic flavor, %	2.94 ^B^	35.3 ^A^	32.4 ^A^	12.1	0.002

Hybrid: hybrid chicken. PAD: Padovana slow-growing breed. POL: Polverara slow-growing breed. ^A,B^ Means in the same row with different superscript letters differ for *p* < 0.01.

**Table 6:** Consumer preference analysis of leg meat according to genotype (Hybrid, PAD, and POL).

Attributes	Genotype			RSD ^1^	*p*-Value
	Hybrid	PAD	POL		
Visual appearance	2.49 ^A^	2.30 ^B^	2.19 ^B^	0.63	<0.0001
Chicken odor	3.05 ^a^	3.03 ^a^	2.86 ^b^	0.77	0.0251
Chicken flavor	3.17 ^A^	2.94 ^Ba^	2.75 ^Bb^	1.18	<0.0001
Juiciness	3.23 ^A^	2.98 ^B^	2.82 ^B^	0.87	<0.0001
Greasiness	3.27 ^Aa^	3.01 ^ABb^	2.81 ^B^	0.89	<0.0001
Overall acceptability	3.26 ^A^	3.03 ^Ba^	2.80 ^Bb^	0.90	<0.0001

Hybrid: hybrid chicken. PAD: Padovana slow-growing breed. POL: Polverara slow-growing breed. ^A,B^ Means in the same row with different superscript letters differ for *p* < 0.01. ^a,b^ Means in the same row with different superscript letters differ for *p* < 0.05. ^1^ Residual standard deviation.

**Table 7:** Consumer preference analysis of leg meat according to age: Students (age <21 years old) and personnel (age > 35 years old).

Attributes	Consumer Age		RSD ^1^	*p*-Value
	Students	Personnel		
Visual appearance	1.93	2.73	0.63	0.0245
Chicken odor	2.65	3.32	0.77	0.0073
Chicken flavor	2.71	3.20	1.18	0.0325
Juiciness	2.67	3.35	0.87	0.0113
Greasiness	2.74	3.32	0.89	0.0532
Overall acceptability	2.71	3.36	0.90	0.0222

^1^ Residual standard deviation.

**Table 8:** Consumer preference analysis of leg meat from Hybrid, PAD, and POL genotypes according to consumer age: Students (age < 21 years old) and personnel (age > 35 years old).

Genotype	Attributes	Consumer Age		RSD ^1^	*p*-Value
		Students	Personnel		
**Hybrid**	Visual appearance	2.19	3.07	1.42	0.004
	Chicken odor	2.78	3.33	1.14	0.026
	Chicken flavor	3.11	3.74	1.21	0.015
	Juiciness	2.99	3.78	1.24	0.004
	Greasiness	3.07	3.57	1.32	0.079
	Overall acceptability	3.12	3.87	1.28	0.006
**PAD**	Visual appearance	1.84	2.70	1.31	0.003
	Chicken odour	2.72	3.27	1.13	0.025
	Chicken flavour	2.71	2.92	1.23	0.428
	Juiciness	2.62	3.23	1.18	0.016
	Greasiness	2.79	3.50	1.27	0.012
	Overall acceptability	2.73	3.55	1.24	0.023
**POL**	Visual appearance	1.74	2.55	1.36	0.063
	Chicken odour	2.52	3.31	1.15	0.002
	Chicken flavour	2.53	3.03	1.29	0.071
	Juiciness	2.50	3.11	1.22	0.018
	Greasiness	2.60	3.18	1.29	0.043
	Overall acceptability	2.45	2.98	1.33	0.063

Hybrid: hybrid chicken. PAD: Padovana slow-growing breed. POL: Polverara slow-growing breed. ^1^ Residual standard deviation.

## Supplementary Materials

The following are available online at https://www.mdpi.com/2076-2615/10/6/960/s1. Table S1: Consumer preference analysis of leg meat according to the gender of consumer: Male (M) and Female (F). Table S2: Effect of frequency of chicken meat consumption on consumer preference analysis.
