# Supplementary material for: Are Meat Quality Traits and Sensory Attributes in Favor of Slow-Growing Chickens?
Source: Animals (Basel). 2020 May 31;10(6):960. doi: 10.3390/ani10060960 (PMC7341280; doi:10.3390/ani10060960)
Supplement: Supplementary file 1 [file animals-10-00960-s001.pdf]

Are meat quality traits and sensory attributes in favour of slow-growing chickens?

Erika Pellattiero, Giulia Tasoniero, Marco Cullere, Elizabeth Gleeson, Gabriele Baldan, Barbara Contiero, Antonella Dalle Zotte

Supplementary Materials

Table S1. Consumer preference analysis of leg meat according to the gender of consumer: Male (M) and Female (F).

	Consumer gender		RSD[1]	*p*-Value
	Male	Female		
Visual appearance	2.39	2.26	0.63	0.5964
Chicken odour	3.13	2.83	0.77	0.0941
Chicken flavour	3.02	2.88	1.18	0.4005
Juiciness	3.05	2.98	0.87	0.6910
Greasiness	2.93	3.13	0.89	0.3733
Overall acceptability	3.07	2.99	0.90	0.7220

[1] Residual Standard Deviation.

Table S2. Effect of frequency of chicken meat consumption on consumer preference analysis.

	Weekly consumption of chicken meat[1]			RSD[2]	*p*-Value
	<1	1-2	>2		
Visual appearance	2.31	2.17	2.51	0.63	0.3396
Chicken odour	2.96	2.91	3.08	0.77	0.5286
Chicken flavour	2.69	3.07	3.09	1.18	0.2369
Juiciness	2.90	3.08	3.06	0.87	0.8083
Greasiness	2.88	3.17	3.04	0.89	0.5415
Overall acceptability	2.82	3.12	3.16	0.90	0.5195

<1: chicken consumption is equal to "less than once a week"; 1-2: chicken consumption is equal to "from one to two times a week"; >2: chicken consumption is equal to "more than twice a week"; [2]Residual Standard Deviation.
